# Effect of experimental periodontitis on cardiac functions: a comprehensive study using echocardiography, hemodynamic analysis, and histopathological evaluation in a rat model

**DOI:** 10.3389/fvets.2023.1327484

**Published:** 2023-12-21

**Authors:** Asmaa Elhaieg, Ahmed Farag, Ahmed Elfadadny, Aimi Yokoi, Hanan Hendawy, Ahmed S. Mandour, Ryou Tanaka

**Affiliations:** ^1^Department of Veterinary Surgery, Faculty of Veterinary Medicine, Tokyo University of Agriculture and Technology, Fuchu, Japan; ^2^Department of Surgery, Anesthesiology, and Radiology, Faculty of Veterinary Medicine, Zagazig University, Zagazig, Egypt; ^3^Department of Animal Internal Medicine, Faculty of Veterinary Medicine, Damanhur University, Damanhour, Egypt; ^4^Department of Veterinary Surgery, Faculty of Veterinary Medicine, Suez Canal University, Ismailia, Egypt; ^5^Department of Animal Medicine (Internal Medicine), Faculty of Veterinary Medicine, Suez Canal University, Ismailia, Egypt

**Keywords:** experimental periodontitis model, alveolar bone, cardiac function, intraventricular pressure gradients, speckle-tracking echocardiography, pressure-volume analysis

## Abstract

**Introduction:**

Periodontitis is a prevalent and severe dental condition characterized by the gradual degradation of the bone surrounding the teeth. Over the past two decades, numerous epidemiological investigations have suggested a potential link between periodontitis and cardiovascular disease. However, the complex mechanistic relationship between oral health issues and cardiovascular disorders remains unclear.

**Aim:**

This study aimed to explore comprehensively the cardiac function through various methods, including conventional echocardiography, intraventricular pressure gradient (IVPG) analysis, speckle tracking echocardiography (STE), and hemodynamics analysis.

**Methods:**

Ligature-induced periodontitis was established in a group of rats while the second group served as sham. The successful establishment of the periodontitis model was confirmed through staining and radiographic examination of the affected mandibles.

**Results:**

X-ray films and methylene blue staining revealed alveolar bone resorption in the affected first molar in the model rats, confirming the successful induction of periodontitis. The rats with periodontitis displayed a decrease in ejection fraction compared to the sham group, accompanied by a decrease in mid-to-apical IVPG and mid IVPG. Lower values of strain rate were recorded in the apical segment of the septum, the middle segment of the septum, and the basal segment of the lateral free wall in the periodontitis group, which was associated with histopathological examination showing some degree of myocardial tissue damage. Conversely, rats with periodontitis showed an increase in heart rate, end-systolic volume, and arterial elastance when compared to the sham rats. However, they also exhibited a decrease in stroke work, stroke volume, cardiac output, and end-systolic pressure.

**Conclusion:**

This study suggests that experimental periodontitis may lead to cardiac dysfunction especially compromised systolic function and myocardial relaxation, potentially indicating an increased risk of cardiovascular events in clinical periodontitis cases. The comprehensive assessment of cardiac function, hemodynamics, and histopathological evaluation underscores the profound impact of periodontitis on heart functions within this specific experimental model.

## Introduction

Cardiovascular disease (CVD) represents a substantial public health concern in various communities, resulting in approximately 17 million fatalities each year (1). In recent times, there has been growing consensus that chronic inflammatory ailments, which induce a widespread inflammatory state, can elevate the susceptibility to CVD alongside traditional risk factors like diabetes, smoking, elevated cholesterol levels, and a lack of physical activity (2–4).

Periodontal disease is a persistent, infectious, and inflammatory ailment arising from an unbalanced subgingival biofilm. As time progresses, it has the potential to lead to the deterioration of the supportive structures around the teeth, encompassing connective attachment loss and the absorption of alveolar bone (5). The progression of periodontal disease is influenced by a multifaceted etiopathology, marked by complex interactions between microorganisms within dental biofilms and the host’s immune-inflammatory response, these microorganisms within dental plaque impact periodontal tissues through both direct and indirect mechanisms, releasing molecules that induce tissue damage and, in turn, initiate the immune-inflammatory response (6).

At the molecular level, cases of active periodontitis exhibit an inflammatory response that can disrupt the overall homeostasis of the individual. This systemic inflammatory reaction may extend to areas beyond the oral cavity (7). Considering the systemic ramifications of periodontal disease, it undeniably wields a substantial influence on overall health, potentially exerting a profound impact on an individual’s quality of life (8, 9).

Remarkably, individuals afflicted with severe chronic periodontitis have shown a significantly heightened risk of developing cardiovascular disease, a correlation that persists even after accounting for various traditional risk factors (10). A meta-analysis involving over 200,000 individuals revealed that periodontal disease (PD) increases the risk of CVD by 35%, underscoring its significant impact on public health (11).

A substantial and increasing body of evidence has established an epidemiological association between periodontal inflammation and cardiovascular diseases, including conditions like arterial hypertension, myocardial infarction, stroke, and atherosclerotic vascular disease (12–14). This relationship has been suggested to occur through both indirect and direct pathways. Indirectly, it may involve shared risk factors that contribute to both periodontitis and cardiovascular diseases (15). On the other hand, a direct mechanism has been postulated, where oral bacteria from periodontal infections can enter the bloodstream, triggering a systemic inflammatory response (16).

Despite the growing evidence supporting the link between periodontitis and cardiovascular disease, the precise mechanisms underpinning this relationship are not yet fully comprehended. Further research is needed to better understand the complex interplay between these conditions and identify the specific pathways involved in their association. Given the limited investigation into the role of periodontal disease in causing cardiovascular dysfunctions, the objective of this study was to evaluate the cardiovascular consequences in a rat model of induced periodontitis. To achieve this goal, various approaches were employed, including echocardiographic assessment of cardiac performance, histopathological examination of myocardium, and hemodynamic recordings. By utilizing these methodologies, the current research aimed to gain insights into the potential impact of periodontitis on cardiac functions in the rat model.

## Materials and methods

### Animal housing and experimental design

The study involved 16 male Sprague–Dawley rats, weighing between 300 and 350 grams and aged 12 to 16 weeks. The experimentation took place in specific laboratories at Tokyo University of Agriculture and Technology. All procedures adhered to the guidelines outlined in the Guide for the Care and Use of Laboratory Animals, as issued by the US National Institute of Health (NIH). The protocols were thoroughly reviewed and approved by the Institutional Animal Care and Use Committee of Tokyo University of Agriculture and Technology (Approval No. R05-159).

The rats were housed in conventional cages, with two rats per cage, and utilized Aspen Shavings as bedding. The housing conditions included an air-conditioned room maintained at (24 ± 2°C), operating on a 12 h light/dark cycle with a relative humidity of 58%. Food and water were provided *ad libitum*, and the rat diets were sourced from the commercial company Oriental Yeast Co., Ltd., based in Tokyo, Japan.

**Figure 1:**
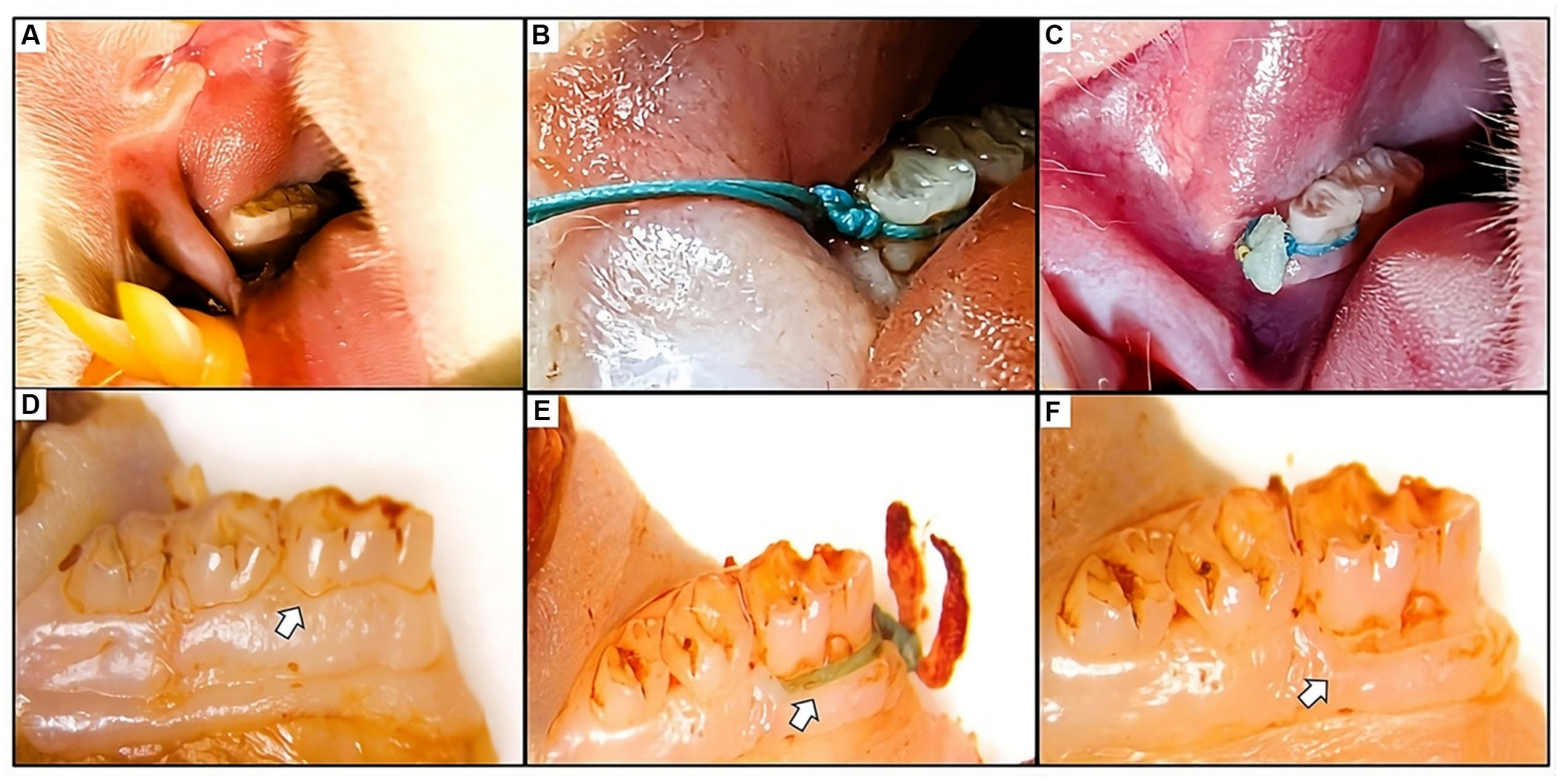
**(A)** Visual representation of the healthy gum tissue before the ligature application. **(B)** Figure depicting the placement of the ligature around the first mandibular molar. Ligatures remained in place throughout the study, with regular assessments and repositioning as needed. **(C)** Clinical observation of the gum tissue at the experiment’s end revealed the presence of the ligature, accompanied by inflammation, food residue, and necrotic tissue. **(D)** Macroscopic images of the molar displaying normal tissue in the sham group. **(E,F)** Macroscopic views of the molar illustrate ligature placement post-soft tissue removal and the resulting alveolar bone loss caused by the ligature around the affected molar.

The rats were divided into two groups, with eight rats in each group: sham operated group and the group with experimentally induced periodontitis (IP). For periodontitis induction, the rats were anesthetized using a combination of medetomidine hydrochloride (Domitor, Orion Pharma Animal Health, Helsinki, Finland), midazolam (Dormicum, Astellas Pharma Inc., Tokyo, Japan), and butorphanol (Vetorphale, Meiji Seika Pharma Co., Ltd.) at the dose rate of 0.3, 5.0, and 5.0 mg/kg body weight, administered subcutaneously, following the completion of all surgical procedures, atipamezole was administered at a dose rate of 1.0 mg/kg subcutaneously to promote a smooth and rapid recovery (17, 18) and the mandibular first molar was ligatured with 3-0 sterile silk sutures as illustrated in Figure 1 (3, 19, 20).

.After 5 weeks of periodontitis induction, the animals underwent anesthesia, followed by echocardiographic and hemodynamic examinations. Subsequently, the rats were euthanized using isoflurane overdose via inhalation, and both mandibles and heart tissues were collected for further analysis.

### Conventional echocardiography

After 5 weeks following surgery, cardiac functions were evaluated in 16 animals (sham and IP groups) one day before euthanasia using an ultrasonographic ProSound 7 system equipped with a 12-MHz transducer supported by CMME and simultaneous ECG from Hitachi-Aloka Medical Ltd., Tokyo, Japan. The echocardiography followed the American Society of Echocardiography (ASE) guidelines (21, 22).

To assess the left ventricle (LV), a two-dimensional right parasternal short-axis view was obtained at the level of the papillary muscles using M-mode. All LV structures were manually measured by the same observer following the leading-edge method of the ASE (22). The recorded values represented the average of a minimum of five consecutive cardiac cycles on the M-mode tracings. Using this perspective, the following parameters were derived: left ventricular internal diameter during diastole (LVIDd), left ventricular internal diameter during systole (LVIDs), left ventricular posterior wall diameter during diastole (LVPWd), left ventricular posterior wall diameter during systole (LVPWs), ejection fraction (EF%), and fractional shortening (FS%). Furthermore, trans-mitral inflow indices, encompassing early (E) and late (A) velocities, along with the E/A ratio, were acquired through pulsed-wave (PW) Doppler echocardiography from the left apical four-chamber view. Tissue Doppler imaging (TDI) was also obtained from the same view. PW TDI echocardiography, utilizing a sample volume of 0.5 mm, was employed to capture the movement of the left ventricular (LV) septal and posterior walls. The TDI velocity profile comprised systolic (s′) and diastolic velocities [early (e′) and late (a′)] at the point of attachment of the mitral valve to the septal and lateral walls of the LV, and these velocities were recorded.

The E/e′ ratio was calculated using the following formula: E/e′ = (E/e′ lateral + E/e′ septal)/2.

### Color M-mode echocardiography

The color M-mode echocardiography (CMME) technique was employed to evaluate the intraventricular pressure gradient (IVPG). To ensure accurate tracing of the continuous mitral valve inflow (CMME), the ultrasound machine was set with a sweep speed of 300 mm/s and a color baseline shift of −64, effectively elevating the Nyquist limit. This configuration enhanced the visualization of the blood flow pathway from the left atrium to the left ventricular (LV) apex through the mitral valve in the left apical four-chamber view. Following this, M-mode was activated to capture the inflow, and color M-mode images were saved for subsequent offline analysis using MATLAB (The MathWorks, Natick, MA, United States).

The calculation of IVPG was carried out using the following formula (23, 24): IVPG (mmHg/cm) = IVPD/LV length.

**Figure 2:**
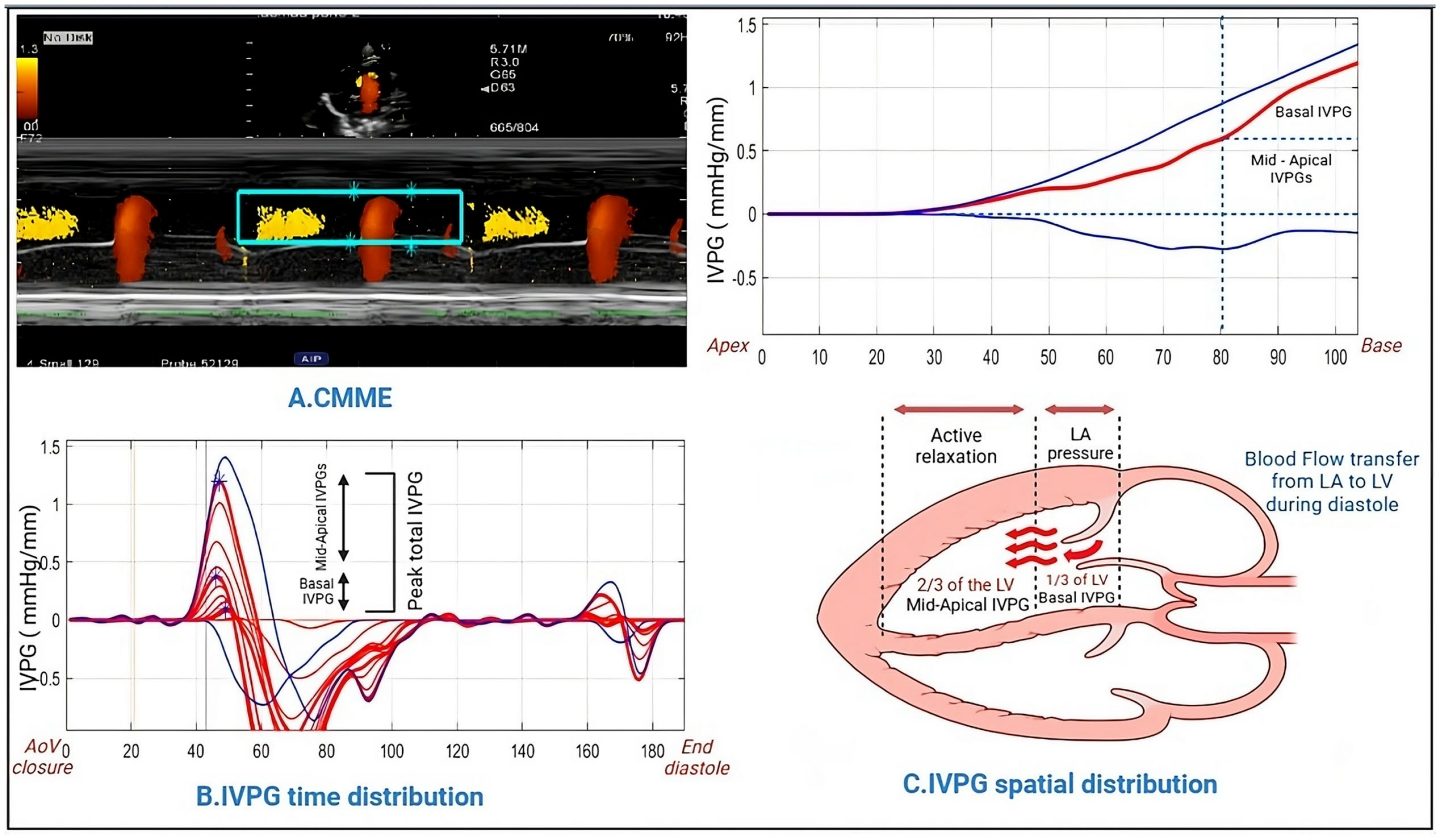
Methodology for IVPG analysis using Matlab. Color M-mode echocardiography was captured and sampled **(A)**, followed by the assessment of time distribution **(B)**. Utilizing Matlab, the spatial distribution of intraventricular pressure gradients was computed, yielding basal IVPG and mid-to-apical IVPG values **(C)**.

The IVPD, commonly characterized as the pressure disparity occurring in the initial phase of diastole between specific segments within the left ventricle (LV), arises when the pressure at the LV apex falls below that at the base (25). The total IVPG was then divided into two segments, based on dividing the LV length into three equal parts. The smaller segment near the mitral valve was termed basal IVPG, while the mid-to-apical segment, covering the other two-thirds near the apex, was considered the other IVPG segment, as shown in Figure 2. For precision and dependability, each data point was measured a minimum of five times at every interval, and the resultant average values were documented and reported for analysis.

### Speckle tracking echocardiography

The study involved acquiring loops of left ventricle (LV) movement from four apical views. To analyze these movements, speckle tracking analysis was conducted using an algorithm incorporated into EchoPAC PC DAS-RSI from Hitachi Aloka Co., Tokyo, Japan.

**Figure 3:**
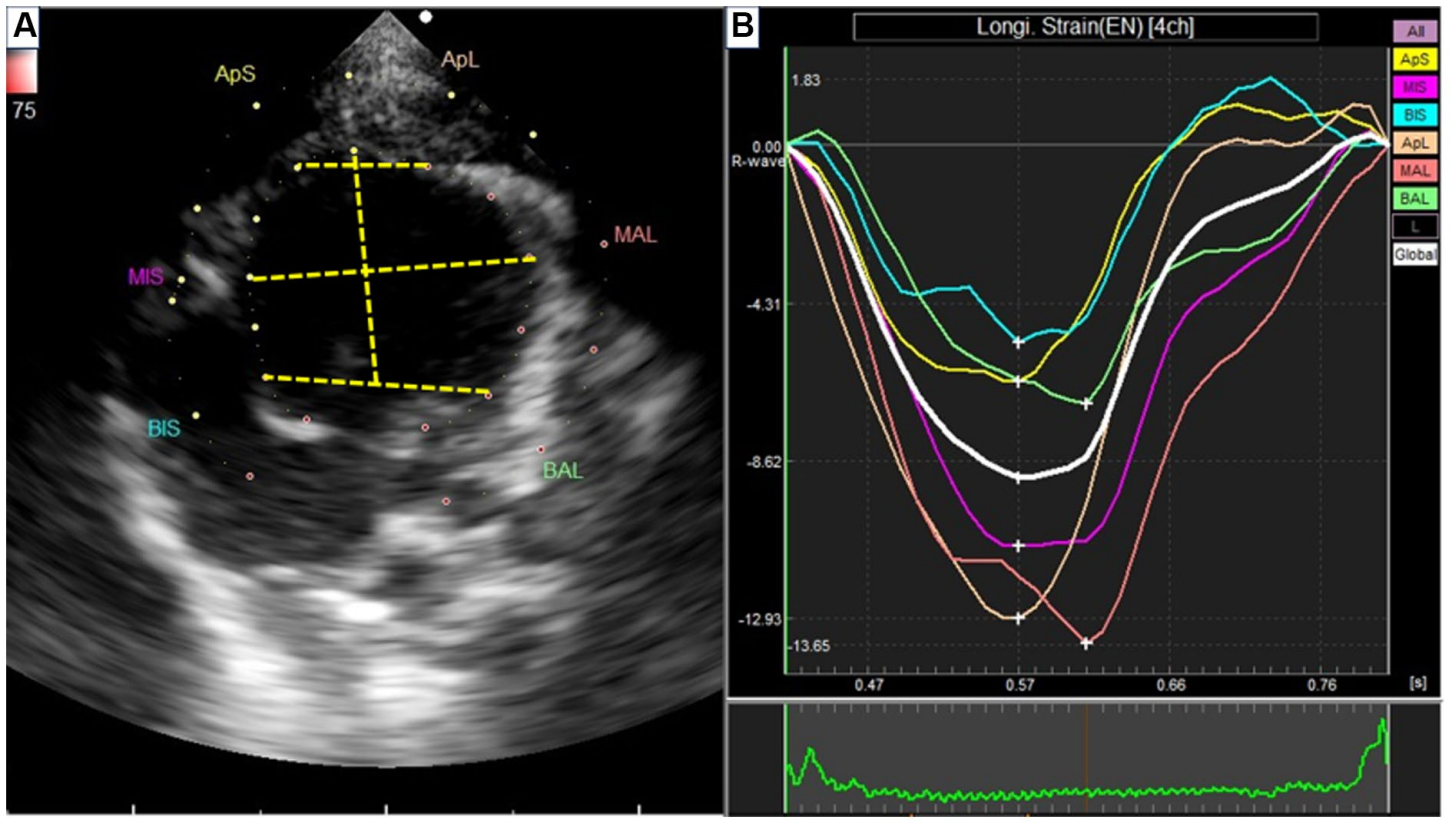
Speckle tracking echocardiography from left apical four-chamber view. **(A)** Segmentation of the left ventricle. APS, the apical segment of the septum; MS, the middle segment of the septum; BS, the basal segment of the segment; APL, the apical segment of the lateral free wall; ML, the middle segment of the lateral free wall; BL, the basal segment of the lateral free wall. **(B)** The strain rate of each segment.

In the analysis process, Manual tracing of the endocardium was performed for both the end-systole and end-diastole phases. Subsequently, the software algorithm automatically segmented each imaging plane of the left ventricle (LV) into three equally circular sections: basal, midventricular, and apex on both the septal and lateral aspects (Figure 3). The longitudinal strain rate was calculated and obtained in six sections for further examination (26). This comprehensive analysis aimed to assess the LV movement and longitudinal strain in different regions of the heart, allowing for a detailed evaluation of cardiac function.

### Hemodynamic measurements

After evaluation of all echocardiographic parameters, pressure and volume measurements were calibrated using the MPVS-Ultra system (Millar Inc., Houston, TX, United States). Anesthetized rats were placed in a supine position on a heated pad. A midline incision along the anterior neck exposed the trachea. Following this, the right carotid artery was dissected, and a Millar catheter was inserted through the artery and guided into the left ventricle (LV) via the aortic valve. After a stabilization period of 5–10 min, pressure-volume (PV) loop signals were continuously recorded at a sampling rate of 1,000 Hz using the MPVS-Ultra Single Segment Pressure-Volume unit (Millar Inc., Houston, TX, United States) (27). Through dedicated PV loop analysis software (Millar Inc., Houston, TX, United States), numerous LV parameters were computed and derived. These encompassed end-systolic volume, LV end-diastolic volume, end-systolic pressure, end-diastolic pressure, the time constant of left ventricular pressure decay (Tau), stroke work (SW), stroke volume (SV), and cardiac output (CO).

**Figure 4:**
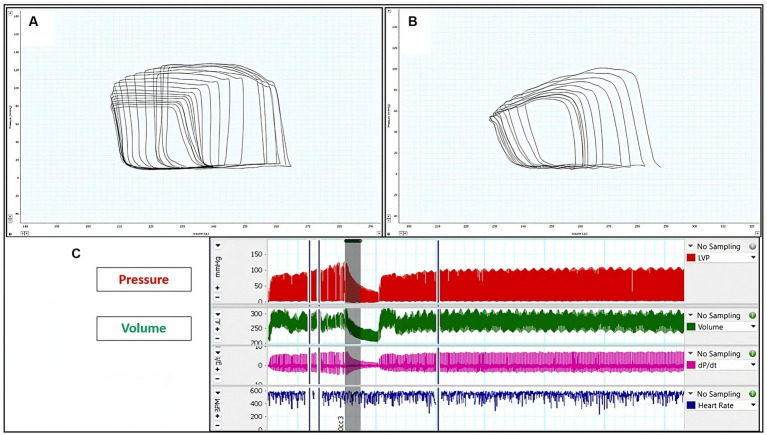
Representative left ventricular (LV) pressure-volume (PV) loops: **(A)** LV PV-loop from sham rats. **(B)** LV P-V loop from rats with induced periodontitis, subsequent to caudal vena cava occlusion. The reduction in pressure signal amplitude within the P-V loop signifies reduced contractility. **(C)** Illustration depicting alterations in pressure and volume following preload reduction achieved by caudal vena cava occlusion.

Furthermore, an assessment of the relationship between LV and PV was undertaken via the occlusion of the caudal vena cava. Essential indices such as the slope of the end-systolic pressure-volume relationship (ESPVR), preload recruitable stroke work (PRSW), and the slope of the end-diastolic PV relationship (EDPVR) were computed to gain insights into the cardiovascular dynamics. Upon the conclusion of each experiment, an intravenous injection of 10 μL of a 15% saline solution was administered to establish a parallel conductance volume. This volume ascertained from the alteration of PV loop relations, was employed to rectify the cardiac mass volume (28, 29). The volume calibration process employed a Millar volume calibration cuvette. All measurements were analyzed using LabChart8 software (ADInstruments, Colorado Springs, CO, United States) (Figure 4).

### Histopathological examination

Following euthanasia through the inhalation method of isoflurane overdose (5%), all animals underwent necropsy. Subsequently, the heart was dissected into smaller fragments and then preserved in a solution of 10% neutral buffered formalin to facilitate fixation. In preparation for analysis, the heart tissue underwent a series of steps: it was sectioned into slices measuring 5 μm using an automated benchtop tissue processor (LEICA TP 1020, Biosystem Tokyo, Japan), followed by deparaffinization and rehydration. The tissue was then subjected to hematoxylin and eosin (H&E) staining, which enabled the assessment of potential inflammatory changes within the cardiac tissue.

To analyze these slides, a light microscope was utilized. To capture these visual insights, image software (CellSens Standard; Olympus, Tokyo, Japan) was employed. Following this, a histopathologist, who remained blind to the experimental groups, assessed 10 sections per group. The analysis encompassed the identification of specific features, including mononuclear cell infiltration, interstitial edema, necrosis, and the arrangement of myocardial cells (whether organized or disorganized and the direction of alignment) (30).

### Evaluation of alveolar bone loss (confirmation of model induction)

#### Methylene blue staining

Macroscopic assessment was employed to evaluate the extent of bone resorption on the lingual and buccal surfaces of the first molars. The specimens were retrieved from alcohol, dried, and subsequently immersed in a solution comprising 0.7 g/L of methylene blue (Sigma, MO, United States) for a duration of 5 min. Excess dye was eliminated by rinsing the samples with tap water (31).

Digital photographs of the lingual and buccal sides of stained first molars were taken from a uniform 90-degree perspective utilizing a stereomicroscope (Leica M60) configured at a 20× magnification level. In these visuals, we conducted an assessment of the region lying between the junction of the cementoenamel and the crest of the alveolar bone on both the buccal and lingual surfaces. This measurement accounts for the entirety of the exposed root surfaces stained in a blue hue, except the enamel on the crown, and was subsequently quantified. This measurement was performed by an examiner who was unaware of the experimental groups, utilizing Image Tool 3.0 software. An increase in the exposed root area, compared to sham, non-ligated teeth, signifies the presence of alveolar bone resorption (20, 31, 32).

#### Radiographic analysis

Radiographs of the affected hemimandibles were taken to evaluate the alveolar bone loss and validate the successful establishment of the animal model. For each rat within the two experimental groups, radiographic assessments were conducted using an X-ray machine (Collimator Type R-20J, Shimadzu Corporation, Japan). The X-ray tube operated at 30 kW, with a current of 6 mA, for 0.01 s, and the distance from the source to the sensor was set at 50 cm. Upon the conclusion of the experiment, the radiographs were utilized to evaluate the dental alveolar bone level, represented by the amount of alveolar bone present and wrapped around the root (33–35).

### Statistical analysis

All statistical analyses were performed using GraphPad Prism 8.0 software (GraphPad Software, San Diego, California). The Mann–Whitney test was utilized for the analysis, and the results are presented as mean ± SD. Differences were deemed statistically significant when the *p*-value was less than 0.05. Spearman’s rank correlation and linear regression analysis were employed to evaluate the relationships between hemodynamic, IVPG, and STE measurements. The coefficient of determination (*R*^2^) was calculated based on the sum of the squares of the distances of the data points from the best-fit curve.

## Results

### Conventional echocardiography

The assessment of cardiac function through conventional echocardiography is presented in Table 1. The findings reveal that in rats with ligature-induced periodontitis, there was a statistically significant increase in LVIDs. Conversely, there were significant reductions in EF% and Sm when compared to the sham group. Nevertheless, parameters such as IVSd, LVIDd, LVPWd, IVSs, LVPWs, FS%, early mitral velocity (E), late mitral velocity (A wave), E/A ratio, Em, Em/Am ratios, and E/Em ratio exhibited comparable values in both groups.

**Table 1:** Assessment of cardiac function using conventional echocardiography.

	Sham group	IP group	*p*-value
IVSd (mm)	1.713 ± 0.112	1.675 ± 0.183	0.96
LVIDd (mm)	8.238 ± 0.702	8.675 ± 0.523	0.08
LVPWd	2.163 ± 0.396	1.988 ± 0.155	0.39
IVSs (mm)	2.275 ± 0.243	2.288 ± 0.28	0.97
LVIDs (mm)	5.288 ± 0.458	5.713 ± 0.419*	0.04
LVPWs (mm)	2.763 ± 0.199	2.475 ± 0.349	0.08
EF %	74.83 ± 2.932	71.11 ± 3.647*	0.04
FS %	36.03 ± 2.100	33.89 ± 2.553	0.06
eV	72.10 ± 5.530	70.84 ± 8.835	>0.99
aV	32.23 ± 3.991	33.10 ± 3.122	0.39
E/A	2.266 ± 0.329	2.066 ± 0.199	0.22
Sm	5.150 ± 0.484	4.575 ± 0.395*	0.02
Em	5.350 ± 0.730	5.300 ± 0.282	0.62
Am	4.213 ± 0.502	4.14 ± 0.551	0.88
Em/Am	1.273 ± 0.127	1.276 ± 0.153	0.89
E/Em	12.09 ± 1.929	13.52 ± 2.286	0.19

Data are presented as the mean ± standard deviation (SD) for each group (*n* = 8). IVSd and IVSs: interventricular septal thickness in end-diastole and systole, respectively. LVIDd and LVIDs: left ventricular diastolic and systolic internal diameter, respectively. LVPWd and LVPWs: left ventricular diastolic and systolic posterior wall thickness, respectively. EF: ejection fraction. FS: fractional shortening. eV: early diastolic transmitral flow velocity. aV: late diastolic transmitral flow velocity. E/A: ratio of early to late diastolic transmitral flow velocities. Sm: left ventricular wall velocity at systole. Em: left ventricular wall velocity at early diastole. Am: left ventricular wall velocity at late diastole. Em/Am: ratio of early to late diastolic velocity of the left ventricular wall. E/Em: ratio of early diastolic velocity mitral to early diastolic velocity of the LV wall.

### IVPG measurements

The findings from the analysis of IVPG data are outlined in Table 2. Notably, the mid-to-apical IVPG in the IP group (0.577 ± 0.047) exhibited a significant reduction compared to the sham group (0.717 ± 0.108). Similarly, the mid IVPG in the IP group (0.515 ± 0.052) was significantly lower than that observed in the sham group (0.637 ± 0.099). Conversely, no statistically significant differences were detected in Total IVPG, Basal IVPG, and Apical IVPG.

**Table 2:** Variability of CMME indices.

	Sham group	IP group	*p*-value
Total IVPG	1.714 ± 0.130	1.635 ± 0.089	0.3282
Basal IVPG	1.047 ± 0.119	1.013 ± 0.103	0.8785
Mid to apical IVPG	0.717 ± 0.108	0.577 ± 0.047*	0.0104
Mid IVPG	0.637 ± 0.099	0.515 ± 0.052*	0.0047
Apical IVPG	0.080 ± 0.038	0.061 ± 0.037	0.2786

Data are presented as the mean ± standard deviation (SD) for each group (*n* = 8). TIVPG, total intraventricular pressure gradient; BIVPG, basal intraventricular pressure gradient; mid-to-apical IVPG, middle-to-apical intraventricular pressure gradient; MIVPG, middle intraventricular pressure gradient; AIVPG, apical intraventricular pressure gradient.

### Speckle tracking echocardiography

Table 3 presents the strain rates obtained through speckle tracking echocardiography. Notably, the IP group exhibited significantly reduced strain rates in specific segments compared to the sham group. Specifically, the apical segment of the septum in the IP group (−3.175 ± 0.696) showed a significant decrease compared to the sham group (−4.663 ± 1.208) with a *p*-value of 0.025. Similarly, the middle segment of the septum in the IP group (−8.411 ± 2.403) displayed a significant reduction compared to the sham group (−13.29 ± 4.009) with a *p*-value of 0.014. Additionally, the basal segment of the lateral free wall in the IP group (−2.811 ± 0.690) exhibited a significantly lower strain rate than the sham group (−7.959 ± 0.689) with a *p*-value of 0.0002. Conversely, in the remaining segments, the IP group demonstrated myocardial movement levels comparable to those observed in the sham-operated rats.

**Table 3:** 2D-speckle tracking echocardiography measurements.

	Sham Group	IP Group	*p*-value
APS	−4.663 ± 1.208	−3.175 ± 0.696*	0.025
MS	−13.29 ± 4.009	−8.411 ± 2.403*	0.014
BS	−11.73 ± 2.504	−11.63 ± 2.261	0.899
APL	−5.782 ± 2.790	−4.338 ± 1.055	0.368
ML	−7.675 ± 1.388	−7.583 ± 1.749	0.823
BL	−7.959 ± 0.689	−2.811 ± 0.690*	0.0002

Data are presented as the mean ± standard deviation (SD) for each group (*n* = 8). APS, strain rate of the apical segment of the septum; MS, strain rate of the middle segment of the septum; BS, strain rate of the basal segment of the septum; APL, strain rate of the apical segment of the lateral free wall; ML, strain rate of the middle segment of the lateral free wall; BL, strain rate of the basal segment of the lateral free wall.

### Pressure-volume loop analysis

Table 4 provides a comprehensive overview of the hemodynamic data in the rats. Notably, the IP group demonstrated significant differences in the majority of parameters when compared to the sham group. Specifically, stroke work, stroke volume, cardiac output, and end-systolic pressure in the IP group exhibited a significant decrease compared to their respective values in the sham group. In contrast, heart rate, end-systolic volume, arterial elastance, and the time constant of left ventricular pressure decay (Tau) in the IP group demonstrated a significant increase compared to their values in the sham group. However, no statistically significant changes were observed in end-diastolic volume and end-diastolic pressure between the two experimental groups.

**Table 4:** Hemodynamic measurements.

	Sham group	IP group	*p*-value
HR (bpm)	301.8 ± 6.573	322.2 ± 1.413	<0.001
SW (mmHg*uL)	3,608 ± 628.6	2,302 ± 751.4	<0.01
CO (uL/min)	8,490 ± 4,057	3,875 ± 148.0	<0.01
SV (uL)	49.66 ± 11.11	31.56 ± 6.708	<0.001
ESV (uL)	241.9 ± 12.18	255.6 ± 7.380	<0.01
EDV (uL)	280.4 ± 11.81	281.2 ± 19.81	0.8785
ESP (mmHg)	119.8 ± 5.928	78.53 ± 10.12	<0.001
EDP (mmHg)	4.326 ± 0.972	5.271 ± 1.726	0.2657
Ea (mmHg/uL)	2.342 ± 0.295	2.845 ± 0.479	0.02
Tau (ms)	19.10 ± 2.253	21.59 ± 1.856	0.047
ESPVR (mmHg/uL)	1.262 ± 0.279	0.880 ± 0.185	0.01
EDPVR	0.032 ± 0.011	0.046 ± 0.028	0.3282
PRSW	99.14 ± 6.771	72.83 ± 12.21	<0.001

The values are presented as means ± standard deviations (SD). HR, heart rate; SV, stroke volume; SW, stroke work; CO, cardiac output; ESV, end-systolic volume; EDV, end-diastolic volume; ESP, end-systolic pressure; EDP, end-diastolic pressure; Ea, arterial elastance, Tau, time constant of left ventricular pressure decay; ESPVR, end-systolic pressure-volume relationship; PRSW, preload recruitable stroke work; EDPVR, end-diastolic pressure-volume relationship.

In Figure 4, representative original P-V loops obtained during the reduction of preload (achieved through transient occlusion of the caudal vena cava) are shown for both sham and IP animals. Functional indices were computed through P-V loop analysis at various preloads during this transient occlusion. Furthermore, it was observed that the end-systolic pressure-volume relationship (ESPVR) was steeper in sham rats compared to IP rats. Conversely, the end-diastolic pressure-volume relationship (EDPVR) showed a tendency to increase in IP rats, although this difference did not reach statistical significance. Moreover, the preload recruitable stroke work (PRSW) values were notably lower in IP rats in comparison to the sham rats.

### Histopathological findings

**Figure 5:**
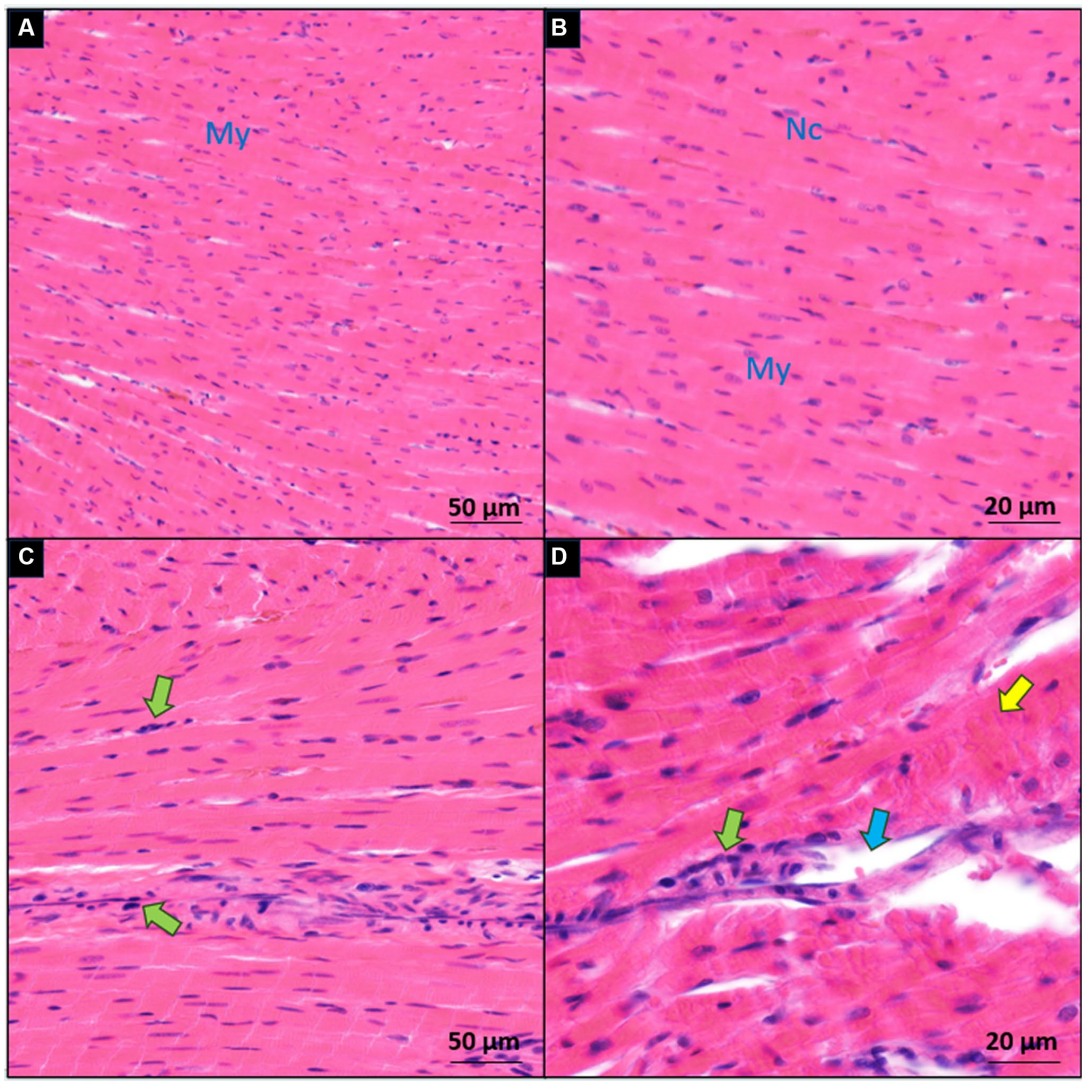
Microscopic comparison of myocardial sections between the sham **(A,B)** and induced periodontitis **(C,D)** groups. Inflammatory cells are highlighted with green arrows, degenerated cells with yellow arrows, and interstitial edema with blue arrows. Cardiac myocytes are denoted as My, and their nuclei as Nc. Histological examination of heart tissue in both groups is presented at two magnification levels [10× for **(A,C)** and 20× for **(B,D)**].

In terms of the histological alterations observed in the hearts of the sham and IP groups, a distinct contrast was evident. The myocardial tissues within the sham group exhibited a normal and unremarkable structure and morphology, as depicted in Figures 5A,B. However, clear histopathological changes were evident in the cardiac tissues of the IP group. These alterations included evident cell degeneration, notable modifications in the shape of cardiac myocytes accompanied by a loss of organized arrangement, and a discernible absence of nucleation. Additionally, there was a noticeable increase in the interstitial tissues, and inflammatory cells were observed around the arterioles, as illustrated in Figures 5C,D. These histological findings substantiate our initial hypothesis. They collectively imply that the observed myocardial tissue damage within the IP group may indeed exert an influence on both the structural integrity and functional aspects of the heart.

### Establishment of the periodontitis model in experimental rats

At the initiation of the experiment, the gingival tissues exhibited a standard appearance characterized by a smooth texture and a light pink hue. The attached gingiva firmly adhered to the underlying structures, while the free gingival margin displayed a distinct outline aligned with the cementoenamel junction (CEJ) of neighboring teeth.

Upon completion of the experiment, notable changes were observed in the clinical state of the gingiva. The macroscopic view showed a cyanotic shift, accompanied by pronounced edema encompassing the observed teeth. Furthermore, the free gingival margin displayed irregularities, including the presence of food debris (Figure 1).

**Figure 6:**
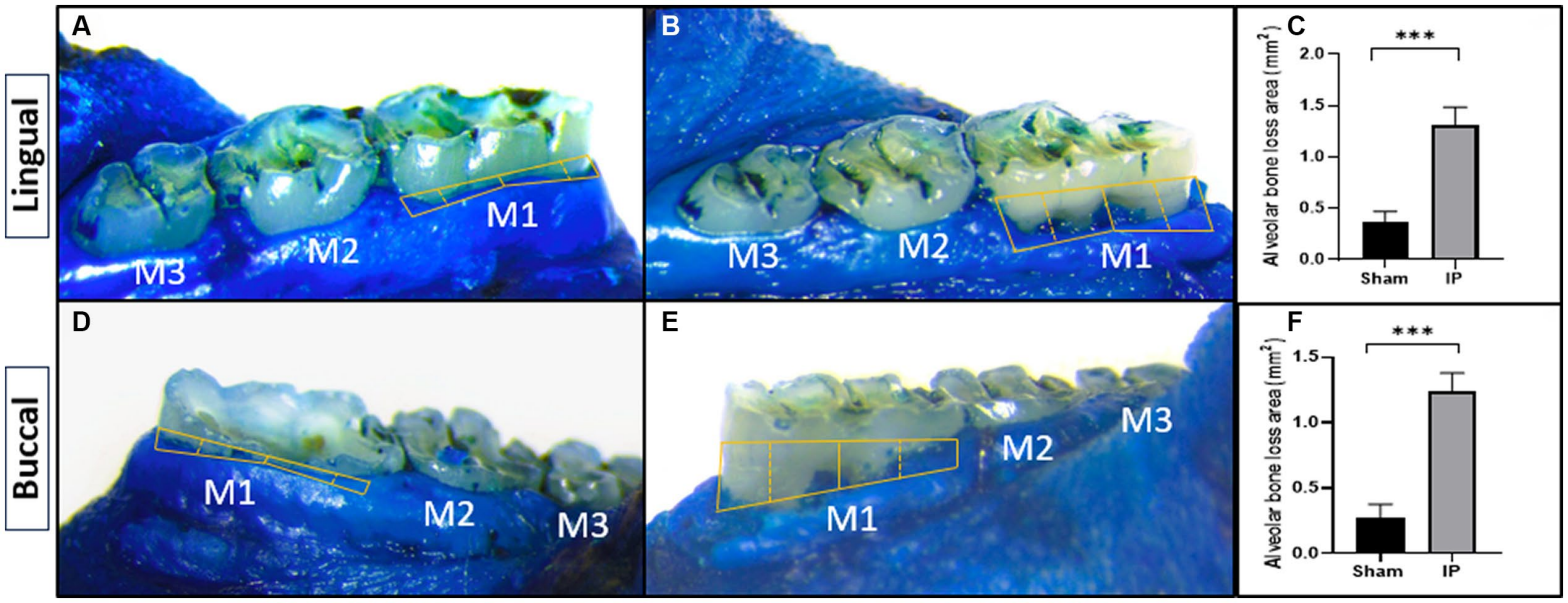
Effect of periodontitis on alveolar bone loss (methylene blue staining). The alveolar bone loss area was macroscopically measured on the lingual **(A,B)** and buccal **(C,D)** surfaces in rats with sham **(A,C)** or induced periodontitis **(B,D)**. Bar graphs show the alveolar bone loss area from the lingual **(E)** and buccal **(F)** surfaces. Yellow solid lines depict the alveolar bone loss region. Data are presented as mean ± standard deviation (SD) ***p < 0.001. IP, induction of periodontitis through ligation of the left mandibular first molar.

Alveolar bone resorption stands as a defining characteristic of periodontitis triggered by the ligature. The contrast between ligature-induced periodontitis rats and the sham group is illustrated in Figure 6, displaying a comprehensive comparison of morphometric measurements. Notably, substantial bone loss is evident on both surfaces, as indicated by significantly increased areas extending from the cementoenamel junction to the alveolar bone crest in ligature-induced periodontitis rats when contrasted with their counterparts that underwent sham procedures (1.313 ± 0.172 vs. 0.362 ± 0.106 for lingual side) and (1.238 ± 0.140 vs. 0.275 ± 0.103 for buccal side).

**Figure 7:**
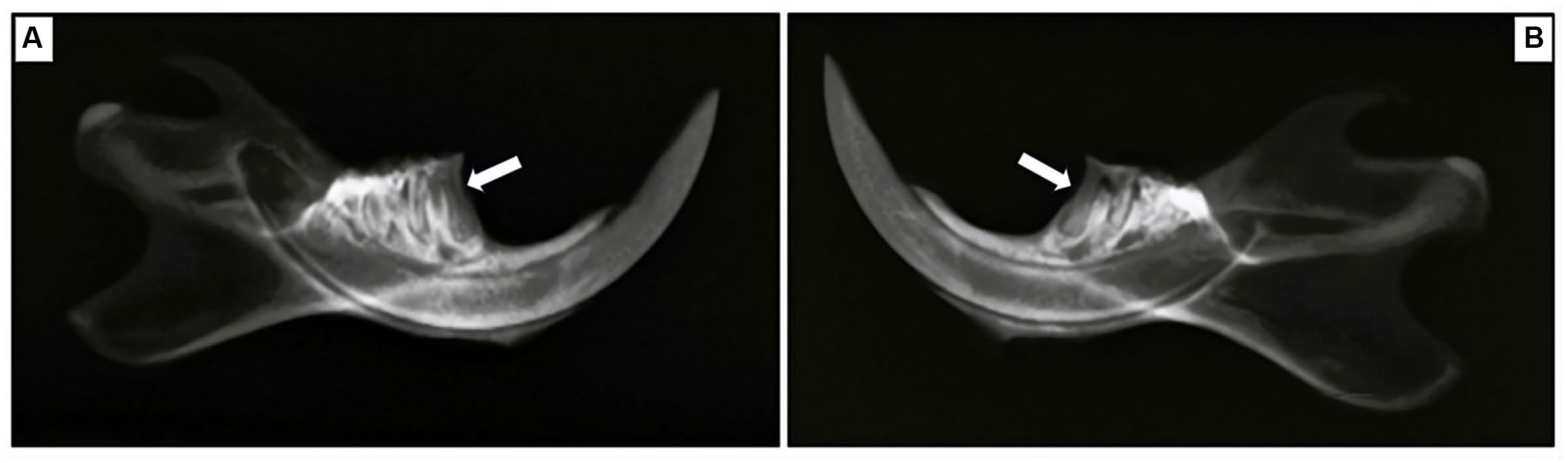
Radiographic exploration of mesial root areas in the first mandibular molar for two groups. **(A)** Sham rats, **(B)** rats with induced periodontitis. Sham rats demonstrate the absence of alveolar bone loss, as the alveolar bone is displayed continuously wrapping around the root, in contrast to their counterparts in the model group. Alveolar bone loss is indicated by the white arrow.

X-ray images revealed evident alveolar bone resorption around the affected molars in the rats with experimentally induced periodontitis, as compared to the sham rats. Notably, the alveolar bone of the sham rats displayed a continuous wrapping around the root, confirming that successful induction of periodontitis had been achieved by the fifth week of the modeling process (Figure 7).

### Correlation and regression analysis between P-V measurements and IVPG indices

Table 5 summarizes the correlation and linear regression analysis results between PV-loop measurements and IVPG indices.

**Table 5:** Correlation and regression analysis between P-V measurements and IVPG indices.

	HR		SW		CO		SV		ESV		EDV		ESP	
	*r*	*R*^2^	*r*	*R*^2^	*r*	*R*^2^	*r*	*R*^2^	*r*	*R*^2^	*r*	*R*^2^	*r*	*R*^2^
Total IVPG	−0.495*	0.245	0.184	0.034	0.491	0.241	0.225	0.05	−0.579*	0.335*	0.155	0.024	0.312	0.097
Basal IVPG	−0.177	0.031	0.107	0.011	0.228	0.052	0.261	0.068	−0.31	0.096	−0.109	0.011	0.178	0.031
Mid-Apical IVPG	−0.611*	0.373*	0.818*	0.67***	0.619*	0.384*	0.326	0.106	−0.305	0.093	0.222	0.049	0.604*	0.365*
Mid IVPG	−0.538*	0.289*	0.85*	0.723***	0.561*	0.315*	0.386	0.149	−0.282	0.079	0.353	0.124	0.588*	0.346*
Apical IVPG	−0.338	0.114	0.115	0.013	0.301	0.091	−0.078	0.006	−0.134	0.017	−0.288	0.082	0.185	0.034

	EDP		Ea		Tau		ESPVR		EDPVR		PRSW	
	*r*	*R*^2^	*r*	*R*^2^	*r*	*R*^2^	*r*	*R*^2^	*r*	*R*^2^	*r*	*R*^2^
Total IVPG	−0.082	0.006	−0.416	0.173	−0.012	0.0001	0.154	0.023	−0.133	0.017	0.266	0.07
Basal IVPG	−0.242	0.058	−0.242	0.058	−0.053	0.002	−0.171	0.029	−0.299	0.089	−0.129	0.016
Mid-Apical IVPG	0.0002	0.0004	−0.539*	0.291*	−0.16	0.025	0.395	0.156	−0.047	0.002	0.617*	0.381*
Mid IVPG	−0.058	0.003	−0.483*	0.233	−0.074	0.005	0.233	0.054	0.11	0.012	0.661*	0.437**
Apical IVPG	0.152	0.023	−0.276	0.076	−0.263	0.069	0.517	0.268*	−0.424	0.18	0.034	0.001

*, **, *** Respectively represent significance *p* < 0.05, *p* < 0.001, and *p* < 0.0001 and are shown in bold.

Significant correlations were found between HR, SW, CO, and ESP with both the mid-apical IVPG and mid IVPG segments (*p* < 0.001, 0.01, 0.01, and 0.01, respectively, for the mid-apical IVPG segment; and *p* < 0.001, 0.02, 0.03, and 0.016, respectively, for the mid IVPG segment). Additionally, Ea and PRSW showed significant correlations with the mid-apical IVPG segment only (*p* = 0.005 and 0.05, respectively).

Similarly, a statistically significant effect of HR, SW, CO, and ESP was observed on both the mid-apical IVPG and mid IVPG segments (*p* = 0.0001, 0.0118, 0.0105, and 0.0132, respectively, for the mid-apical segment; and *p* < 0.0001, 0.0237, 0.0314, and 0.0164, respectively, for the mid IVPG segment). Moreover, there was a significant effect of EDV and ESP on mid and mid-to-apical IVPG.

### Correlation and regression analysis between P-V measurements and STE parameters

Table 6 summarizes the correlation outcomes between P-V measurements and STE parameters. It also presents the coefficient of determination (*R*^2^) derived from linear regression analysis, indicating the extent of its impact on the corresponding parameters.

**Table 6:** Correlation and regression analysis between P-V measurements and STE parameters.

	HR		SW		CO		SV		ESV		EDV		ESP	
	*r*	*R*^2^	*r*	*R*^2^	*r*	*R*^2^	*r*	*R*^2^	*r*	*R*^2^	*r*	*R*^2^	*r*	*R*^2^
ApS	0.501*	0.251*	−0.255	0.065	−0.379	0.143	−0.499*	0.249*	0.32	0.102	0.07	0.004	−0.66**	0.443**
MIS	0.573*	0.328*	−0.384	0.147	−0.231	0.053	−0.577*	0.333*	0.409	0.167	−0.034	0.001	−0.542*	0.294*
BIS	0.078	0.006	−0.143	0.02	−0.056	0.003	−0.058	0.003	−0.073	0.005	−0.299	0.089	0.011	0.0001
ApL	0.329	0.108	−0.342	0.117	−0.312	0.097	−0.034	0.001	0.1	0.01	0.245	0.06	−0.354	0.125
MAL	−0.084	0.007	−0.331	0.11	0.005	0.00002	−0.151	0.022	0.039	0.001	−0.092	0.008	−0.073	0.005
BAL	0.872***	0.761***	−0.694**	0.482**	−0.621*	0.385*	−0.757***	0.573***	0.513*	0.263*	−0.101	0.01	−0.908	0.824***

	EDP		Ea		Tau		ESPVR		EDPVR		PRSW	
	*r*	*R*^2^	*r*	*R*^2^	*r*	*R*^2^	*r*	*R*^2^	*r*	*R*^2^	*r*	*R*^2^
ApS	0.284	0.08	0.353	0.125	0.522*	0.272*	−0.629**	0.396**	0.479*	0.229	−0.34	0.115
MIS	0.164	0.026	0.541*	0.293*	0.442*	0.196	−0.395	0.156	0.323	0.104	−0.552*	0.305*
BIS	0.029	0.0008	0.001	0.0001	−0.201	0.04	0.106	0.011	−0.279	0.078	−0.393	0.154
ApL	−0.101	0.01	0.238	0.057	0.519*	0.269*	−0.346	0.119	0.222	0.049	−0.119	0.014
MAL	−0.298	0.088	0.038	0.001	0.057	0.003	0.31	0.096	−0.454	0.207	−0.187	0.035
BAL	0.402	0.161	0.604*	0.365*	0.47	0.221	−0.639**	0.408**	0.346	0.12	−0.856***	0.734***

*, **, *** Respectively represent significance *p* < 0.05, *p* < 0.001, and *p* < 0.0001 and are shown in bold.

HR exhibited significant correlations with APS, MIS, and BAL segmental regions (*p* = 0.04, 0.02, and <0.001, respectively). SW, CO, and ESV were also correlated with the BAL segment (*p* = 0.002, 0.01, and 0.041, respectively). Additionally, SV showed significant correlations with APS, MIS, and BAL segmental regions (*p* = 0.04, 0.01, and 0.0006, respectively). Furthermore, Ea correlated with MIS and BAL (*p* = 0.03 and 0.01), while Tau correlated with APS, MIS, and ApL regions (*p* = 0.03, 0.08, and 0.03, respectively). ESPVR exhibited correlations with APS and BAL (*p* < 0.01 for both), and PRSW correlated with MIS and BAL regions (*p* = 0.02 and <0.001).

Moreover, HR, SV, and ESP demonstrated significant effects on APS, MIS, and BAL segmental regions (*p* = 0.04, 0.04, and 0.004, respectively, for APS; 0.02, 0.01, and 0.02, respectively, for MIS; and <0.001 for all for BAL segmental region). Ea had a significant effect on MIS and BAL (*p* = 0.03 and 0.0132), while Tau exhibited a significant effect on APS and APL (*p* = 0.03 for both). ESPVR showed significant effects on APS and BAL (*p* < 0.01), and PRSW also had significant effects on MIS and BAL (*p* = 0.02 and <0.001).

## Discussion

In this study, the bone loss seen in rats subjected to dental ligation indicated that the examined model of periodontitis was straightforward. Through the evaluation of cardiac function (using echocardiography), hemodynamics (via PV loop analysis), and histopathological examination of myocardial tissue structure, the impact of periodontitis on the cardiovascular system was revealed in this experimental periodontitis model.

In this study, we employed an anesthetic protocol involving a combination of MMB and atipamezole. Numerous studies on the use of MMB in mice and rats have been documented (18, 36–38). Beyond these species, the anesthetic efficacy of MMB has been explored in other laboratory animals, including monkeys (39), cotton rats (40), and hamsters (41).

According to Kirihara et al. (42), following the administration of MMB, a reduction in blood pressure was observed until the 20 min mark, after which it stabilized during anesthesia. MMB, acting as an alpha2-adrenergic receptor agonist, is known to decrease blood pressure (43). However, Kirihara et al. (42) noted a significant increase in systolic blood pressure at 10 min post-administration in the MMB group compared to the KX group. This contradictory effect may be attributed to MED’s specificity as an alpha2-adrenergic receptor agonist in comparison to XYL (44). The elevated blood pressure at 10 min post-MMB administration suggests MED’s interaction with alpha2B receptors, causing a temporary peripheral vessel constriction (45). Baumgartner et al. (46) also reported a transient increase in blood pressure with the anesthetic mixture of MED, mid, and fentanyl, attributed to MED’s initial peripheral vasoconstrictive properties. Notably, this temporary rise in blood pressure post-MMB administration was documented in dogs (47) but not in monkeys (48) and mice (42).

Kirihara et al. (42) further observed a decrease in heart rate from approximately 240 beats/min to around 170 beats/min for the initial 20 min post-MMB administration, with stability thereafter. The heart rate gradually increased after the surgical anesthesia duration, with no significant differences between MMB and KX groups. In a separate investigation, it was documented that following the administration of the MMB anesthetic mixture, the respiratory rate in the SD strain was lower compared to that observed after saline administration. Conversely, the respiratory rates in the WST and F344 strains did not show any significant difference following the administration of the anesthetic mixture compared to saline. Subsequent to the administration of the anesthetic mixture, the respiratory rates across the three distinct rat strains became nearly identical and remained stable for the duration of the experiment (49).

### Top of form

Alveolar bone loss stands out as a significant characteristic of periodontitis. This deterioration of bone structure results from a complex interplay of immune and inflammatory processes, as our body seeks to combat oral bacterial dysbiosis (50). The outcomes of our study illustrated that the bone loss detected in rats undergoing dental ligation, as evidenced by both methylene blue staining and radiographic analysis, faithfully recreated the periodontitis model. These findings are consistent with earlier research, which affirms the successful establishment of a periodontitis model through the induction of alveolar bone resorption (20, 31, 51).

The conventional echocardiographic analysis showed a significant reduction in ejection fraction and a decrease in fractional shortening, although the fractional shortening did not reach a statistical significance level. This suggests a potential compromise in left ventricular systolic function in rats with ligature-induced periodontitis. This finding is in line with Ribeiro and colleagues’ research (20), which demonstrated that experimental periodontitis leads to cardiac dysfunction, elevated cardiac cytokines, and sympathetic overactivity. These results align with epidemiological studies that indicate an elevated risk of cardiovascular events in clinical periodontitis cases. Furthermore, several studies have highlighted the correlation between the severity of periodontitis and cardiac dysfunction in human subjects (52, 53). On the other hand, in our study, most of the conventional echocardiographic parameters were not found to be statistically significant between the two experimental groups. This suggests that conventional echocardiographic examination may not be suitable for detecting subtle changes in systolic and diastolic function, emphasizing the need for the use of other advanced examination methods such as IVPG and STE analysis.

Echocardiography is a prevalent diagnostic tool in cardiovascular research trials and clinical settings (54). Furthermore, there is a growing interest in noninvasive diagnostic tools. Currently, IVPG stands out as a reliable, noninvasive, preload-independent, and highly precise indicator of diastolic function. This is particularly pertinent in the evaluation of cardiac structure and function in cases of myocardial pathology. It is calculated by dividing the intraventricular pressure decay (IVPD) by the left ventricular (LV) length (25). Our study findings underscore a decrease in IVPG (specifically Mid, and Mid-to-apical regions) in rats with ligature-induced periodontitis. Earlier research by Courtois et al. (55) investigating IVPG determinants showcased significant reductions in IVPG during acute coronary occlusion, along with a correlation between diminished IVPG and extensive regional systolic dysfunction. This, combined with their prior research, contributed to their hypothesis concerning the interaction between IVPG and the left ventricle’s (LV) elastic recoil. This suggests a mechanism that supports LV filling at lower diastolic pressures. Impairments in regional systolic function could lead to reduced energy release during diastole, subsequently resulting in abnormal or decreased intraventricular flow. In addition, our hemodynamic measurements, especially end-systolic volume (ESV), support this hypothesis. In animals with periodontitis, ESV increased, these findings are in accordance with Steine et al. (56), who compared results from color M-mode echocardiography with invasively acquired pressure gradients. In their study, they observed a decrease in IVPG along with an inverse relationship with end-systolic volume (ESV). However, it remains uncertain from these findings whether isolated changes in systolic function, such as ESV, can manifest independently of alterations in diastolic function, or if these two properties are interconnected through the elastic recoil properties of the LV (57).

Speckle-tracking echocardiography operates by tracing the movement of speckle patterns generated by the interference of ultrasound beams within the myocardium over time (26). This technique captures the degree and velocity of deformation within a particular myocardial area in relation to its initial dimensions. One of the key advantages of speckle-tracking echocardiography lies in its ability to evaluate cardiac function across both long and short-axis viewing planes, enabling the assessment of longitudinal, radial, and circumferential strain for any selected area of interest. This method has proven valuable in detecting ischemic regions, particularly in studies related to myocardial infarction (58, 59). Moreover, it holds promise for advancing our comprehension of regional cardiac dysfunction within chronic disease conditions (60).

While the application of speckle tracking echocardiography as a tool for *in vivo* assessment of myocardial strain in mice was introduced over 15 years ago (61), only a limited number of studies have utilized this technique to examine specific measures of cardiac function in rodents. In our study, the regional analysis of longitudinal strain rate unveiled noteworthy findings, particularly within the apical and middle segments of the septum, as well as the basal segment of the lateral free wall. These regions appear particularly susceptible to diastolic dysfunction caused by periodontitis, suggesting that they could potentially serve as indicators of early systolic impairment in the longitudinal plane.

The examination of pressure-volume loops (PV loops) in hemodynamics has emerged as the widely accepted method for studying intricate, *in vivo* cardiac function. This approach facilitates the concurrent measurement of pressure and volume signals in the beating hearts of intact rodents. On one hand, PV-loop analysis has significantly enhanced our understanding of molecular cardiac physiology by facilitating the identification of crucial functional relationships. On the other hand, it permits the examination of the cardiovascular effects of particular therapeutic interventions or specific signaling pathways through the use of transgenic disease models (62). Regarding hemodynamic measurements, we observed tachycardia in rats with periodontitis, which aligns with the findings of Ribeiro and colleagues (20), lending support to the concept of sympathetic overactivation. It is widely recognized that persistent sympathetic overactivity is linked to the development of organ damage, including cardiac hypertrophy and compromised kidney function (63). Within this context, the outcomes of our present study bolster the notion of an elevated cardiovascular risk associated with gingival infection in the evaluated model especially compromised systolic function and myocardial relaxation.

Both load-dependent parameters (including stroke volume, stroke work, and cardiac output) and load-independent parameters (such as ESPVR and preload recruitable stroke work) derived from pressure-volume (P-V) loops indicated a gradual decline in left ventricular (LV) function in the periodontitis rat models. This decline is evidenced by a reduction in the amplitude of the P-V loops, signifying waning contractility in the affected hearts. The overall deterioration in LV function seems to stem from an initial substantial decline in systolic function (manifested as reduced end-systolic pressure—ESP, SV, CO, and SW). In addition, throughout the cardiac cycle, the heart’s work operates within the boundaries delineated by the End-diastolic pressure-volume relationship (EDPVR) and the ESPVR. Within the normal physiological range of LV systolic and diastolic pressures, ESPVR remains relatively unaffected by preload and afterload variations, making it a dependable marker of LV contractility. Notably, the slope of the ESPVR was significantly reduced in the periodontitis rat models. This trend, in conjunction with other contractility measures (such as PRSW), clearly indicates a subtle deterioration in contractility and subsequent systolic function (64).

Our investigation also encompassed an examination of the impact of induced periodontitis on the left ventricular muscle tissue, with a specific focus on myocyte morphology, histopathological assessments following 5 weeks of induced periodontitis revealed a range of noteworthy observations. These included instances of inflammatory cell infiltration, myocyte degeneration, and a loss of organized arrangement. Furthermore, interstitial changes were also noted. Notably, comparable studies employing similar experimental designs have reported substantial inflammatory cell infiltration in various cardiac tissues. For instance, IP was found to induce significant inflammatory cell infiltration in myocardial tissue (19), the aortic wall (4), and the atrium (65).

The research conducted by Köse et al. (19) provided insights into the early chronic phase effects of periodontitis on heart tissue, revealing degenerative and hypertrophic changes. Furthermore, the authors posited that extended exposure to systemic inflammatory stress might elevate the risk of hypertrophic alterations. Miyajima et al. (4) demonstrated that periodontitis triggers the adherence of monocytes and macrophages to aortic endothelial cells, achieved through an increase in p65 NF-kΒ-mediated vascular cell adhesion molecule-1 expression. This adhesion mechanism is postulated to also apply to ventricular endothelial cells, potentially influencing muscle tissue. The consequential release of a multitude of cytokines and growth factors by these adhered cells is believed to contribute to the histopathological changes mentioned earlier. In another study, Yu et al. (65) discovered hypertrophy, particularly in left atrial myocytes, during histopathological evaluations conducted 90 days after exposure to experimental periodontitis in dogs. Notably, this hypertrophy was not significantly observed in ventricular myocytes. From this perspective, it is plausible to consider that histopathological changes in heart tissue due to periodontitis entail degenerative changes during the initial phase, which potentially transition to hypertrophic changes during the chronic stage, dependent on the extent of exposure to inflammatory stress.

## Conclusion

The study highlights the potentially significant role of periodontitis in causing cardiovascular dysfunction. Comprehensive cardiac assessments showed subtle changes in cardiac parameters, demonstrating how periodontal inflammation may functionally compromise CVD progression. Advanced cardiac evaluation techniques such as pressure gradient analysis, speckle tracking, and PV loop analysis were employed to emphasize the importance of using cutting-edge methods to understand complex biological relationships. This research marks a crucial step in comprehending the intricate links between periodontal health and cardiovascular well-being, with the potential to advance clinical practices and improve patient outcomes in these interconnected domains.

### Limitations

While our study has yielded valuable insights, it is important to acknowledge certain limitations. We did not elucidate the intricate mechanistic pathways responsible for the observed cardiac changes, potentially overlooking crucial components that contribute to the link between periodontitis and cardiac dysfunction. This omission underscores the need for further investigations into the molecular underpinnings of this relationship. Nevertheless, our research has provided valuable non-invasive data on cardiac function and structure. This data serves as a foundation for future research endeavors aiming to delve deeper into the molecular intricacies of this connection and the relationship between the severity of periodontitis and the degree of impairment in heart function requires further research.

## Data availability statement

The original contributions presented in the study are included in the article/supplementary material, further inquiries can be directed to the corresponding authors.

## Ethics statement

The animal study was approved by the Tokyo University of Agriculture and Technology’s Institutional Animal Care and Use Committee (Approval No. R05-159). The study was conducted in accordance with the local legislation and institutional requirements.

## Author contributions

AsE: Data curation, Formal analysis, Methodology, Visualization, Writing – original draft. AF: Data curation, Formal analysis, Methodology, Investigation, Writing – original draft. AhE: Methodology, Visualization, Writing – original draft. AY: Formal analysis, Visualization, Writing – original draft. HH: Formal analysis, Visualization, Writing – original draft. AM: Investigation, Writing – review & editing. RT: Investigation, Methodology, Supervision, Visualization, Writing – review & editing.
